# Effects of food variability on growth and reproduction of *Aedes aegypti*

**DOI:** 10.1002/ece3.1888

**Published:** 2016-01-09

**Authors:** Michael Zeller, Jacob C. Koella

**Affiliations:** ^1^ Institute of BiologyUniversity of NeuchâtelRue Emile‐Argand 112000NeuchâtelSwitzerland

**Keywords:** *Aedes aegypti*, compensatory growth, diet restriction, life‐history evolution

## Abstract

Despite a large body of knowledge about the evolution of life histories, we know little about how variable food availability during an individual's development affects its life history. We measured the effects of manipulating food levels during early and late larval development of the mosquito *Aedes aegypti* on its growth rate, life history and reproductive success. Switching from low to high food led to compensatory growth: individuals grew more rapidly during late larval development and emerged at a size close to that of mosquitoes consistently reared at high food. However, switching to high food had very little effect on longevity, and fecundity and reproductive success were considerably lower than in consistently well‐fed mosquitoes. Changing from high to low food led to adults with similar size as in consistently badly nourished mosquitoes, but even lower fecundity and reproductive success. A rapid response of growth to changing resources can thus have unexpected effects in later life and in lifetime reproductive success. More generally, our study emphasizes the importance of varying developmental conditions for the evolutionary pressures underlying life‐history evolution.

## Introduction

How life histories respond to variation in food availability is a central question of evolutionary ecology. Considerable effort, both with theoretical and empirical approaches, has been spent on answering the question for environments that vary spatially (Kawecki and Stearns 1993; Ernande et al. 2004) and from one generation to the next (Bashey 2006) in resource availability. Yet, an important aspect of variability has received considerably less attention: that resource levels can vary during an individual's development. Even though there is substantial evidence that variation in food levels during development can affect age and size at maturity (e.g., Leips and Travis 1994; Hentschel and Emlet 2000), we know little about how this variation affects reproductive success and adult survival.

As food restriction severely affects life‐history parameters – it generally slows growth, delays maturity and leads to small adults with low fecundity (Stearns and Koella 1986) – it seems plausible that individuals that grow slowly early in life should try to make up their size deficit with compensatory growth, that is by growing more rapidly or for a longer period once they obtain more food (Dmitriew 2011). Rapidly growing individuals, on the other hand, might respond to food stress by decelerating growth rates in order to use the available resources for maintenance and reproduction. Slow growth has shown to be adaptive for dealing with nutrient stress (Arendt 1997).

Compensatory growth following a period of unfavorable environmental conditions has been described for many vertebrates and invertebrates (Dmitriew 2011). However, compensatory growth need not be evolutionarily beneficial. Indeed, the presumed benefit of compensatory growth – larger individuals have greater fecundity – is not always observed. In Trinidadian guppies, for example, compensatory growth is not associated with increased, but with decreased fecundity (Auer et al. 2010). Furthermore, any benefit of compensatory growth with regard to fecundity may be counteracted by costs with regard to other parts of the life history. Longer growth and thus delayed maturity, for example, can be associated with a greater risk of dying before maturity (Abrams and Rowe 2007). Even for pure compensatory growth, that is when maturity is not delayed, the greater growth rate may have costs (physiological/cellular level), which are often only evident much later in life (Metcalfe and Monaghan 2001; Alonso‐Alvarez et al. 2007; De Block and Stoks 2008). Indeed, diet restriction is often associated with a longer and healthier life (Chippindale et al. 1993; Masoro 2005). Accordingly, compensatory growth in fish reduced lifespan whereas decelerated growth extended it (Lee et al. 2013). This may, in part, be due to developmental errors and structural instability as a result of increased growth (Mangel and Munch 2005).

Thus, although the role of refeeding after a period of dietary restriction (and, more generally, the role of changing resource availability during an individuals' development) on traits such as growth rate, longevity and age at maturity have acquired some attention, little is known about its role on reproductive success.

In this study, we provide data on the effect of variability in developmental food conditions (leading to compensatory growth or decelerated growth) and associated changes in longevity and reproductive success of the mosquito *Aedes aegypti*. Such data not only form the basis for our understanding of life‐history evolution, but are also directly relevant for public health due to mosquito's role as a vector of several infectious diseases.

## Materials and Methods

### Experimental system

We used the UGAL strain of the mosquito *A. aegypti* (obtained from Patrick Guérin, University of Neuchâtel). *Aedes aegypti* occurs throughout the tropics and subtropics. During the aquatic larval stages, mosquitoes in nature can experience periods of nutrient restriction and competition for resources like bacteria, algae and organic matter (Reiskind and Lounibos 2009).

### Experimental design

The experiment was run in a climate chamber set to 26°C, 70% relative humidity and at 12 h light and 12 h dark regime.

We used a 2 × 2 factorial design, where larvae were fed either with a standard amount of food (Day 1: 0.06 mg of tetramin fish food, day 2: 0.08 mg, day 3: 0.16 mg, day4: 0.32 mg, day 5: 0.64 mg, day 6 or later: 0.32 mg) or with half of the standard diet during either early (0–3 days after hatching) or late development (4 or more days after hatching). The four treatments are hereafter referred to as LL, LH, HH, and HL, with the first letter referring to the amount of food during early development (low or high) and the second letter to the amount of food during late development.

Eggs were hatched in deionized water. Four hours after hatching, 384 first instar larvae were moved into 12‐well plates and kept individually in 3 mL of deionized water. Each larva was haphazardly assigned to one of the four feeding regimes and fed every 24 h with the appropriate amount of food. Pupae were moved to 300‐mL plastic cups containing deionized water and a piece of filter paper as an oviposition substrate. The cups were covered with mosquito netting, and cotton wool moistened with 10% sugar solution was placed onto the netting and changed every 48 h. One day after emergence, males were discarded and each female was given a male chosen haphazardly from our colony. The next day and every 10 days thereafter, the females were given the opportunity to take a blood meal on MZ's arm for 5 min. The females where checked every day for survival. Nine days after blood feeding, the females were placed into freshly prepared plastic cups and their eggs were removed and counted. Fecundity was defined as the number of melanized eggs laid up to 9 days after blood feeding. The experiment was stopped after six rounds of egg‐laying, at which time 85.4% of the mosquitoes had died.

### Trait measurement

We estimated larval body size by taking standardized digital pictures of all individuals every 24 h starting on the day of hatching (age 0) and measuring the length of the larva with the open‐access software IMAGEJ. When photos of larvae were considered too low in quality for an accurate measurement to be taken, the individuals were not included in the analyses. Larval growth was measured as the difference in size between age 0 and age 4 (early growth) and between age 4 and age 6 (late growth) for all individuals. The size of adults was assayed as the mean of their wing length, which strongly correlates with the weight of mosquitoes (Koella and Lyimo 1996) and is widely used as an approximation for adult size. The wings were removed and mounted on microscope slides. The slides were digitally scanned and the wings were measured with IMAGEJ (Rasband [Link]).

### Statistical analysis

We considered only females, and ignored the growth of the six (of 384) individuals that had died before pupation. We assayed 185 female mosquitoes, between 43 and 49 in each food treatment.

The difference in size between age 0 and age 4 (early growth) was evaluated with an analysis of variance (ANOVA) that included the level of early food as a fixed binomial factor. Because the size differences between the ages 4 and 6 (late growth) were close to linear and individuals not yet reached asymptotic size they were evaluated with an analysis of covariance (ANCOVA) that included early and late food, the interaction between the two as fixed factors, and the size at age four as a covariate. As size at age four did not interact with early or late food, we omitted these interactions from the analysis. Additionally, because we measured individuals repeatedly, we checked that the results were similar, when we corrected for regression to the mean (analysis not shown). For both analyses (early and late growth), we verified that the assumptions of ANOVA and, respectively, ANCOVA were not violated. Age at emergence and longevity was analyzed with survival analyses that included early and late food and their interaction as fixed factors. In the analysis of longevity, we added wing length as a potential confounder. We used the distributions that gave the best fit, so log‐logistic distribution for age at emergence and Weibull for longevity using proportional hazards gave similar results (not shown). Wing length was analyzed with an ANOVA that included early food and late food and their interaction as fixed factors. The wing lengths were Box–Cox transformed to meet ANOVA requirements.

We analyzed fecundity in three ways. First, we analyzed the proportion of blood‐feeds that led to at least one egg with a GLM (binomial distribution). Second, we analyzed the total number of eggs laid throughout the experiment with a GLM with quasi‐Poisson distribution (corrected for overdispersion). In both analyses, we included early and late food and their interaction as fixed factors and wing length as a potential confounder. Third, we analyzed the age‐specific clutch sizes (considering only those blood‐feeds after which at least one egg had been laid) with a mixed‐effect ANOVA, using early food, late food, clutch number (i.e., age) and their interactions as fixed factors, wing length as a potential confounder, and mosquito as a random effect. We present the analysis using all clutches. As the number of mosquitoes surviving to the end of the experiment was low, we verified that the results were similar if we considered only the first three or the first four clutches (analyses not shown).

The mixed‐effect ANOVA was done with R v.0.98.1056 (R Development Core Team, 2015) using the lme4 package; the other analyses were done with JMP 12.0.0 (JMP^®^, Version 12.0.0, SAS Institute Inc., Cary, NC, USA).

## Results

### Developmental traits

**Figure 1:**
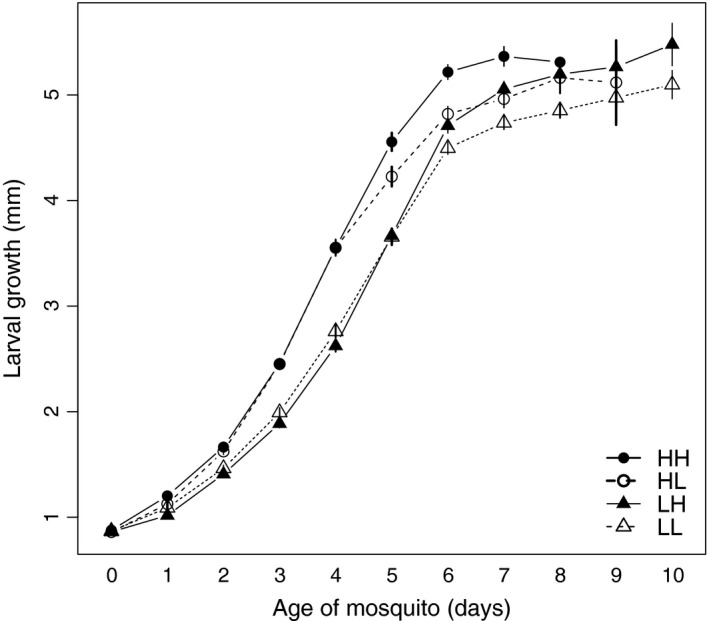
Body length for mosquito larvae as a function of age. Symbols represent the means with each food treatment, vertical lines the standard errors. Triangles represent treatments with low food availability during early development; circles represent treatments with high food availability in early development. Open symbols represent treatments with low food during late development; solid symbols represent high food during late development.

**Figure 2:**
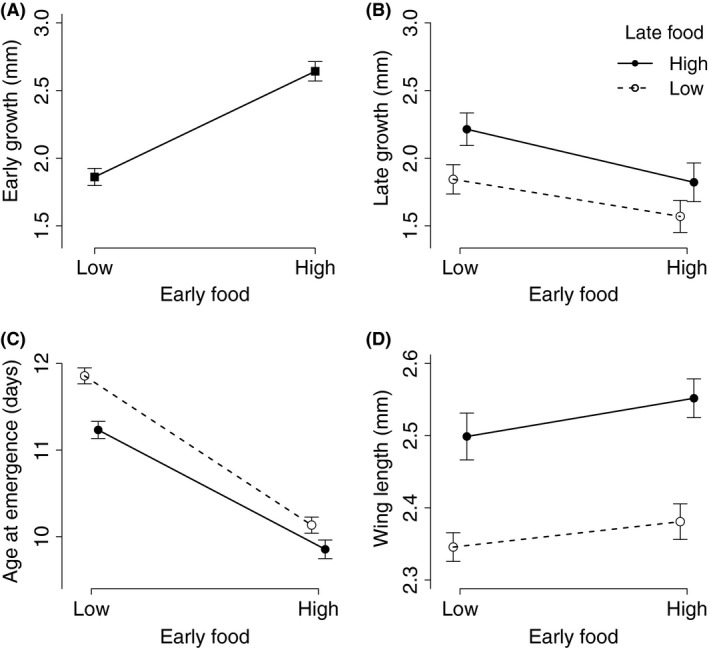
The effect of larval food during early and late stages of development for (A) Early growth (size difference between age 0 and age 4), (B) Mean late growth (size difference between day 4 and day 6), (C) Age at emergence ±SE, (D) Adult size (wing length). The data for early growth (A) were pooled for late food treatment. Symbols represent the means within treatments; the vertical lines their standard errors. Open symbols represent treatments with low food during late development; solid symbols represent high food during late development.

The growth data are summarized in Fig. 1. Larvae reared on high food grew more between age 0 and age 4 (mean = 2.64 mm, standard error = 0.072) than those reared on low food (mean = 1.86 mm, SE = 0.063) (*F* = 66.86, *P* < 0.001) (Fig. 1). Growth after age 4 decreased with increasing size at day 4 (Table 1). It was greatest for mosquitoes that switched from low to high food at age 4 (2.21 mm, SE = 0.120), lowest for mosquitoes that had switched from high to low food (1.57 mm, SE = 0.118), and intermediate for mosquitoes with the same food level throughout their development (Fig. 2B). The effects of early and of late food, but not the interaction between the two, were statistically significant (Table 1).

**Table 1:** Statistical summary for juvenile traits. ANCOVA for differences in late growth, survival analysis (log‐logistic distribution) for age at emergence and ANOVA for differences in wing length

Factor	Late growth				Age at emergence			Wing length			
	df	*F*	SS	*P*	df	*χ* ^2^	*P*	df	F	SS	*P*
Early food	1	5.07	1.77	0.026	1	173.6	<0.001	1	2.84	0.08	0.094
Late food	1	14.87	5.18	<0.001	1	25.5	<0.001	1	41.21	0.02	<0.001
Early food × late food	1	0.24	0.08	0.63	1	6.6	0.01	1	0.13	<0.01	0.721
Size at age 4	1	90.62	31.57	<0.001							
Error	155		52.61					166		4.64	

Age at emergence increased from 9.9 days (SE = 0.11) for mosquitoes consistently fed the high food level to 11.9 days (SE = 0.09) for mosquitoes consistently fed the low food level (Fig. 2D). Mosquitoes that had switched from high to low food emerged earlier (10.1 ± 0.09) than those that had switched from low to high food (11.2 ± 0.10); the interaction between early and late food levels was statistically significant (Table 1, Fig. 2C).

Wing length increased from a mean of 2.35 mm (SE = 0.019) for mosquitoes that had been consistently reared on low food to 2.55 mm (SE = 0.027) for mosquitoes that had been consistently reared on high food. Wing length was influenced significantly by the availability of food after age 4, while early food and the interaction between early and late food had no significant effects (Table 1, Fig. 2D).

### Adult traits

Adult mosquitoes lived longest if they had been reared on low food throughout their development (39.1 days ± 1.97; this and other averages are biased, for the experiment was stopped when 14.6% of the mosquitoes were still alive), followed by those that had switched from low food to high food when they were 4 days old (36.8 days ± 2.44). In contrast to the size of adult mosquitoes, longevity was significantly affected by early food (Table 2), while late food and the interaction between the two food levels had no significant effects. Wing length had no significant effect on longevity (Table 2).

**Table 2:** Statistical summary for adult traits. Survival analyses (Weibull distribution) for longevity, binomial GLM for the proportion of blood‐feds, and GLM (quasi‐Poisson distribution) for the total number of eggs

Factor	df	Longevity		Egg‐laying after blood feeding		Total number of eggs	
		*χ* ^2^	*P*	*χ* ^2^	*P*	*χ* ^2^	*P*
Early food	1	3.87	0.049	0.74	0.39	0.03	0.857
Late food	1	0.22	0.636	4.58	0.032	11.79	<0.001
Early food × late food	1	<0.01	0.969	4.00	0.046	3.66	0.055
Wing length	1	1.17	0.279	0.14	0.712	0.06	0.803

**Figure 3:**
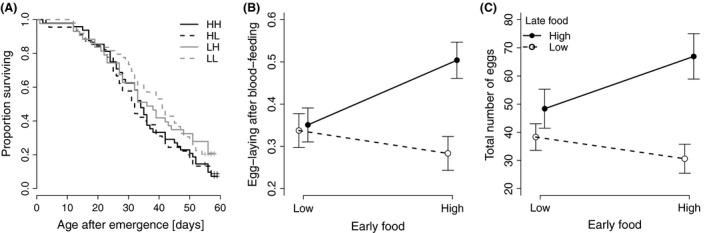
The effect of larval food during early and late stages of development for (A) Longevity of adult female mosquitoes (age 0 is age after emergence), (B) Proportion of blood‐feds that led to egg‐laying, (C) Total number of eggs ± SE. LL stands for low food availability during the whole larval development, LH for low food during early development, high food during late development, HH for high during the whole development and HL for high food during early development, low food during late development. In (B) and (C), open symbols represent treatments with low food during late development; solid symbols represent high food during late development.

The percentage of the six blood‐feeds that were followed by laying at least one egg ranged from 0% to 100%; the average percentage ranged from 50% for the mosquitoes that had been reared on high food throughout their development to 28% if the mosquitoes had switched from high food to low food when they were 4 days old (Fig. 3B). About 35% of the blood‐feeds led to egg‐laying, if mosquitoes had initially been reared on low food, independently of the food available to them during their late development (Table 2). Similarly, the total number of eggs was highest for mosquitoes that had been reared on high food throughout their development (67 ± 8.1), lowest for mosquitoes that had switched food from high to low (31 ± 5.2), and intermediate for mosquitoes that had been reared on low food early in their development (for LL: 38 ± 4.7; for LH: 48 ± 6.9) (Table 2, Fig. 3C). Late food environments had significant effects in determining the probability of laying eggs and the total number of eggs. The interaction between early food and late food had marginally significant effects in determining egg‐laying success and marginally nonsignificant effects in determining the total amount of eggs. Neither the egg‐laying success nor the number of eggs was significantly influenced by wing length (Table 2).

**Figure 4:**
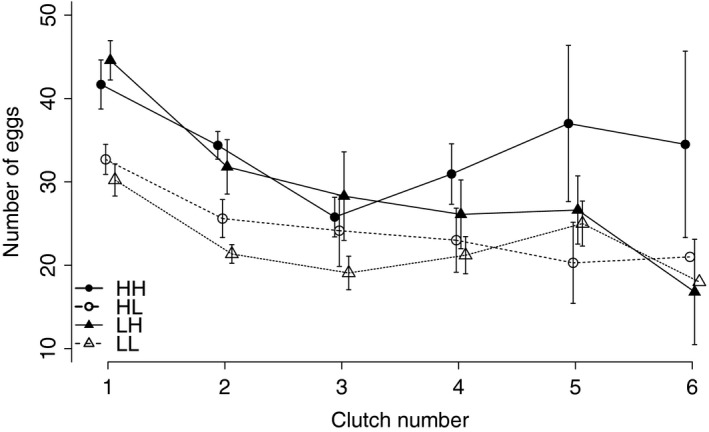
Relationship between number of eggs per clutch and clutch number (i.e., age). Circles represent treatments with high food availability during early development; triangles represent treatments with low food availability in early development. Open symbols represent treatments with low food during late development; solid symbols represent high food during late development.

The clutch size (considering only those blood‐feeds after which at least one egg had been laid) decreased with the age of adult mosquitoes (Fig. 4). Food level during late larval life affected the number of eggs in the first clutch and the rate at which fecundity decreased with age was influenced by the interaction between early and late food treatment (Table 3). Switching from low food to high food 4 days after hatch led to the most eggs in the first clutch, but then to the greatest decline over clutches (Fig. 4). The rate of the decrease was mostly influenced by the interaction between early and late food treatments (Table 3).

**Table 3:** Repeated measures analysis of clutch sizes. Only blood‐feeding attempts, which led to at least one egg, were considered

Factor	Number of eggs			
	df	*F*	SS	*P*
Early food	1	0.02	2.8	0.885
Late food	1	15.00	2005.0	<0.001
Early food × Late food	1	3.67	490.6	0.056
Wing length	1	0.15	19.8	0.700
Clutch number	1	33.81	4518.5	<0.001
Clutch number × Early food	1	0.29	38.3	0.593
Clutch number × Late food	1	0.61	81.8	0.435
Clutch number × Early food × Late food	1	5.52	737.6	0.020
Error	267		133.6	

## Discussion

Variability in developmental food conditions in *A. aegypti* had qualitatively different effects on the life‐history traits we investigated: adult size, fecundity, survival and reproductive success. Thus, for example, wing length was determined mainly by food availability during late larval development, survival by food availability during early development, and total number of eggs by a combination of the two.

When food availability was held constant during the mosquitoes' development, their life history followed the general predictions of life‐history theory (e.g., Stearns and Koella 1986): low food thus led to slow growth, late pupation, small adults, and low fecundity. It also corroborates many studies where food restriction increased longevity (Weindruch 1996; Shanley and Kirkwood 2000; Mair et al. 2003; Kirkwood and Shanley 2005; Masoro 2005).

Varying food availability led to life histories that are more difficult to explain with life history, similar to the study of Yearsley et al. (2004). Increasing from low to high food led, as frequently observed (Metcalfe and Monaghan 2001), to compensatory growth: at emergence, mosquitoes that had been first badly and then well nourished caught up in size by growing more rapidly and by delaying pupation, and thereby became almost as large as mosquitoes that had been fed well throughout their development. However, although size caught up, we observed no to very little catching up of fecundity, longevity, or life‐time reproductive success. Together with the observation that the number of eggs per clutch declined strongest with age for individuals that had switched from low to high food during development (Fig. 4), these results could mean that compensatory growth early in life is associated with reproductive costs later in life, which lead to, in our laboratory conditions, lower life‐time reproductive success. In addition to considerable evidence for trade‐offs between life‐history traits early and late in life, both from laboratory situations (e.g., Rose 1984) and, more recently, from natural populations (Lemaître et al. 2015), our results support the findings of Auer et al. (2010), which suggest that there are reproductive costs associated with compensatory growth. The trade‐off we observed raises the question about the adaptive nature of compensatory growth. However, although in our laboratory conditions, compensatory growth had a negative consequence for reproductive success, the situation may change in natural conditions. Both juvenile and adult mortality rates may be substantially higher in the field than in the laboratory. Accordingly, the benefits of larger size and earlier maturity associated with compensatory growth may outweigh its reproductive costs in old mosquitoes.

When mosquitoes started out at good food conditions and then switched to low food, their growth and adult size decreased as expected. What was more surprising was that the individuals with decelerated growth have lower reproductive success than those that had experienced food restriction throughout their development. However, because the interaction between early and late food was marginally not significant, we cannot draw strong conclusions. Nevertheless, this trend could be the result of physiological responses to the food environment in early development that prepare the individual for a similar environment later in life (Gluckman and Hanson 2004). Therefore, mosquitoes that are undernourished early in life can cope with food restriction later in life better than those that have been prepared for an environment with plentiful food.

A striking result was that wing length had very little effect on reproduction or longevity, although associations of life‐history traits with size are central to many ideas in life‐history theory (e.g., Stearns and Koella 1986; Rowe and Ludwig 1991; Abrams and Rowe 2007). For example, most models that predict the evolutionarily optimal age at maturity assume that fecundity increases with body size (e.g., Roff 1984; Stearns and Koella 1986; Berrigan and Koella 1994). Such associations are often found when food availability is held constant (Lyimo and Takken 1993; McCann et al. 2009). However, in our experiment, where food availability varies during the mosquito's development, the environmental factor over two time‐periods that determined body size (food availability during early and during late development) affect the life‐history traits rather than body size itself. If this is generally the case, it would imply major changes in the way we think about life‐history evolution.

The timing of resource restriction during development also affected its effect on longevity. We observed only an effect if the restriction was during early development. This is consistent with the common finding that food restriction can slow the aging process (Weindruch 1996; Shanley and Kirkwood 2000; Mair et al. 2003; Kirkwood and Shanley 2005; Masoro 2005). However, that changing from low to high or from high to low food had negligible effects on longevity contradicts other studies showing that compensatory growth associated with better food conditions reverses the effect of early resource restriction on longevity (Merry 2002; Dhahbi et al. 2004; Spindler 2005). We have no explanation for the difference of these results.

## Conclusion

In conclusion, we showed that variability of developmental food conditions in *A. aegypti* mosquitoes has strong effects on adult size, reproductive success and mortality of adult females, with some traits being mostly affected by the food availability in early development and other being affected by late food availability. Such effects may have important consequences for energy allocation strategies, but are generally not considered in model of life‐history evolution. We further showed that compensatory growth, which is generally considered an adaptive strategy, does not increase its reproductive success, at least for *A. aegypti* in our laboratory conditions. The reproductive burdens associated with compensatory growth may play an important and limiting role in the evolution of growth and other related traits. Finally, that the mosquitoes' reproductive success was not directly connected with adult size, but was, rather, influenced by the food conditions that they experienced during development contrasts a central assumption of many ideas in life history theory. Thus, we suggest that our understanding of the evolution of life histories will be greatly enhanced if we consider the effects of varying the environmental conditions during juvenile development. Such information is important in order to develop effective predictions of disease transmission and strategies of mosquito control.

## Conflict of Interest

None declared.
